# Assessment of miR-103a-3p in leukocytes—No diagnostic benefit in combination with the blood-based biomarkers mesothelin and calretinin for malignant pleural mesothelioma diagnosis

**DOI:** 10.1371/journal.pone.0275936

**Published:** 2022-10-14

**Authors:** Carmina Jiménez-Ramírez, Daniel Gilbert Weber, Guadalupe Aguilar-Madrid, Alexander Brik, Cuauhtémoc Arturo Juárez-Pérez, Swaantje Casjens, Irina Raiko, Thomas Brüning, Georg Johnen, Alejandro Cabello-López

**Affiliations:** 1 Laboratorio de Análisis Clínico, Unidad Médica de Alta Especialidad, Hospital de Traumatología “Dr. Victorio de la Fuente Narváez”, Instituto Mexicano del Seguro Social (IMSS), Mexico City, Mexico; 2 Institute for Prevention and Occupational Medicine of the German Social Accident Insurance, Institute of the Ruhr University Bochum (IPA), Bochum, Germany; 3 Facultad de Medicina, Departamento de Salud Pública, Universidad Nacional Autónoma de México (UNAM), Mexico City, Mexico; 4 Unidad de Investigación de Salud en el Trabajo, Centro Médico Nacional Siglo XXI, IMSS, Mexico City, Mexico; Institute for Bioscience and Biotechnology Research, ITALY

## Abstract

Malignant pleural mesothelioma (MPM) is a cancer associated with asbestos exposure and its diagnosis is challenging due to the moderate sensitivities of the available methods. In this regard, miR-103a-3p was considered to increase the sensitivity of established biomarkers to detect MPM. Its behavior and diagnostic value in the Mexican population has not been previously evaluated. In 108 confirmed MPM cases and 218 controls, almost all formerly exposed to asbestos, we quantified miR-103-3a-3p levels in leukocytes using quantitative Real-Time PCR, together with mesothelin and calretinin measured in plasma by ELISA. Sensitivity and specificity of miR-103-3a-3p alone and in combination with mesothelin and calretinin were determined. Bivariate analysis was performed using Mann-Whitney *U* test and Spearman correlation. Non-conditional logistic regression models were used to calculate the area under curve (AUC), sensitivity, and specificity for the combination of biomarkers. Mesothelin and calretinin levels were higher among cases, remaining as well among males and participants ≤60 years old (only mesothelin). Significant differences for miR-103a-3p were observed between male cases and controls, whereas significant differences between cases and controls for mesothelin and calretinin were observed in men and women. At 95.5% specificity the individual sensitivity of miR-103a-3p was 4.4% in men, whereas the sensitivity of mesothelin and calretinin was 72.2% and 80.9%, respectively. Positive correlations for miR-103a-3p were observed with age, environmental asbestos exposure, years with diabetes mellitus, and glucose levels, while negative correlations were observed with years of occupational asbestos exposure, creatinine, erythrocytes, direct bilirubin, and leukocytes. The addition of miR-103a-3p to mesothelin and calretinin did not increase the diagnostic performance for MPM diagnosis. However, miR-103a-3p levels were correlated with several characteristics in the Mexican population.

## Introduction

Malignant pleural mesothelioma (MPM) is a lethal cancer of the pleura caused by exposure to asbestos fibers, which is considered a group I carcinogen by the International Agency for Research on Cancer (IARC) and has been banned in more than 60 countries worldwide [1,2].

Three different MPM subtypes exist: epithelioid (the most frequent), sarcomatoid, and biphasic [3]. MPM shows a latency period of 20 to 50 years after asbestos exposure and is usually diagnosed at later stages of the disease, due to non-specific symptoms and moderate sensitivity of current diagnostic methods [3,4]. Moreover, response to current treatments is poor, and thus, survival is commonly low. Nonetheless, the combination of ipilimumab and nivolumab showed an increased survival of patients with diagnosed MPM [5]. Currently, diagnosis of MPM is based on histopathology and immunohistochemistry (IHC), despite the low sensitivity of these methods [3,4]. Therefore, it has been proposed that certain combinations of non-invasive biomarkers might improve MPM diagnosis [6–9]. Among those, mesothelin and calretinin showed promising results [6]. Mesothelin is a 41 KDa glycoprotein present in mesothelial cells derived from the *MSLN* gene, which also encodes megakaryocyte potentiating factor (MPF) [10]. Elevated plasma mesothelin levels have been reported among MPM cases in several studies, which placed this biomarker as the most prominent biomarker for MPM diagnosis [11]. However, mesothelin alone has a relatively low sensitivity [10,12–14]. On the other hand, its combination with calretinin, a 29 kDa calcium-binding protein that is also found in mesothelial cells and functions as a diagnostic biomarker of MPM, has already been evaluated [6,8,15]. In this sense, Jiménez-Ramírez et al. reported a sensitivity of 82.7% in men and 86.8% in women of a Mexican population when both molecules were used jointly for MPM diagnosis [6,8]. The combination of calretinin and mesothelin was additionally shown to be feasible for the early detection of mesothelioma using plasma samples of mesothelioma patients up to 15 months prior to MPM diagnosis [15]. Yet, there is still room for improvement by including additional biomarkers to this evaluated combination.

In this regard, microRNAs (miRNAs) have also gained interest in the diagnosis of several diseases, including MPM. MicroRNAs are non-coding RNA molecules of about 22 nucleotides (nt), which can be determined in the bloodstream (plasma, serum, and leukocytes). These miRNAs regulate several biological processes such as cell differentiation, proliferation, and apoptosis, through imperfect pairing with messenger RNA (mRNA), thus under- or over-expression can occur in different health or disease conditions [12,16,17]. Furthermore, miR-16, miR-132-3p, miR-103a-3p, miR-548a-3p, miR-20, miR-16, miR-17, miR-126, miR-486, miR-548a-3p, miR-20a, miR-486, miR-625-3p, miR-32-3p, miR-197-3p and miR-1281, have been proposed as likely non-invasive plasma biomarkers for MPM [18–24]. Mainly miR-103a-3p, which can be detected in the cellular fraction of blood, might be a promising candidate biomarker for MPM diagnosis due to its increased sensitivity for MPM diagnosis in combination with mesothelin [25]. Testing the effectiveness of these biomarkers is crucial for populations with current asbestos exposure–despite being proven as human carcinogen, such as in Mexico, where more than 500 MPM cases occur each year since 2010, and an underreporting of 71% has been estimated, causing an MPM epidemic [26,27]. Therefore, the aim of this study was to evaluate miR-103a-3p in leukocytes, together with mesothelin and calretinin in plasma, as an additional biomarker for MPM diagnosis. Likewise, the role of clinical and sociodemographic variables that could modify miR-103a-3p levels in a Mexican population was explored.

## Methods

A total of 108 cases and 218 controls, matched by sex and age (± one year), were analyzed in this study, including 82 cases and 212 controls from a previous study by Jiménez-Ramírez et al. [6]., and 32 new participants (26 cases and 6 controls). Cases were defined as patients who attended medical examinations at the Mexican Social Security Institute (IMSS) and the National Institute of Respiratory Diseases (INER) in Mexico City, who had a confirmed MPM diagnosis by IHC. Controls were recruited from the National System of IMSS beneficiaries (SINDO)–a registry for retirees, and the National Institute for the Elderly (INAPAM)–an elderly general population registry [9]. Each participant signed an informed consent form prior to recruitment in the study. Socio-demographic data, detailed history of asbestos exposure, biochemical parameters such as glucose, creatinine, cholesterol, triglycerides, and a complete blood count were included. This project was approved by IMSS’ National Scientific and Ethical Research Commission, with registration number R-2011-785-069, and by INER’s Science and Bioethics in Research Committee with the registration number C30-12.

### Blood samples collection

Six milliliters (mL) of venous blood were obtained using EDTA tubes and centrifuged at 2,500 xg for 10 minutes, within 30 minutes after blood collection. Plasma and leukocyte fraction were separated and frozen immediately at -70°C.

Aliquots of plasma and leukocytes were shipped to Germany under stringent frozen conditions for determination of calretinin and miR-103a-3p.

### Determination of mesothelin and calretinin

Mesothelin and calretinin were measured in plasma using commercial enzyme-linked immunosorbent assays (ELISA) for mesothelin (DY3265, R&D Systems, Minneapolis, MN) and calretinin (DLD Diagnostika GmbH, Hamburg, Germany) according to manufacturer’s instructions. All samples were analyzed in duplicate, and a 5% coefficient of variation was allowed.

### Determination of miR-103a-3p

RNA was isolated from 100 μl leukocytes using the NucleoSpin miRNA kit (Macherey-Nagel GmbH & Co KG, Düren, Germany) according to manufacturer’s instructions. Subsequently, miR-103a-3p was determined as described elsewhere with miR-125a as reference using quantitative Real-Time PCR (qRT-PCR) [19]. Most of the samples (N = 275) were analyzed in 2015 using the 7300 Real-Time PCR System (Thermo Fisher Scientific, Darmstadt, Germany), whereas 51 samples were analyzed in 2021 using the QuantStudio 7 Pro PCR system (Thermo Fisher Scientific, Darmstadt, Germany). Differences between cases and controls were similar in 2015 and 2021 (S1A Fig). Although group differences between years exist, all miR-103a-3p levels measured in 2021 were within the range of the measurements from 2015 (S1B Fig). Indeed, Matias-Garcia et al. reported that miR-103a-3p is not altered by long-term frozen storage or freeze-thaw cycles [28]. Thus, all miR-103a-3p values were integrated in subsequent analyses.

### Statistical analysis

As previously reported [6], biomarker levels differ between males and females. Consequently, biomarker concentrations were analyzed separately according to sex. Biomarker concentrations were reported as medians and interquartile range (IQR). Mann-Whitney *U*, Chi-squared or Fisher’s exact test were used to compare medians and proportions between groups, considering an alpha level of 0.05. Spearman’s rank correlation coefficients were reported between variables. Receiver operating characteristics (ROC) curves were constructed to calculate the area under the curve (AUC) for each biomarker, sensitivities were calculated at fixed specificities for each individual biomarker and in combination. Stata 14 SE (StataCorp LLC, TX, USA) and GraphPad Prism 6 (GraphPad Software, La Jolla, CA, USA) were used for analyses and graphical presentation of data.

## Results

The main characteristics of the study group are depicted in Table 1. In summary, 90 MPM cases and 179 controls were men, and 18 cases and 39 controls were women. Median age of the subjects was 62 years in cases and controls. Epithelioid mesothelioma was the most common subtype accounting for 94.4% (N = 102) of all cases. For most cases (96.3%; N = 104) and controls (91.3%; N = 199), a former exposure to asbestos (occupational or environmental) was recorded.

**Table 1:** Main characteristics of malignant pleural mesothelioma cases and healthy controls by gender in a Mexican population.

Variables	Total sample		Men		Women	
	Cases N (%)	Controls N (%)	Cases N (%)	Controls N (%)	Cases N (%)	Controls N (%)
Total sample	108 (33.1)	218 (66.9)	90 (33.5)	179 (66.5)	18 (31.6)	39 (68.4)
Age (years) [Median (IQR)]	62 (55–71)	62 (55–71)	63 (55–71)	62 (55–71)	60 (53–68)	61 (53–68)
≤60 years	48 (44.4)	96 (44.0)	39 (43.3)	77 (43.0)	9 (50.0)	19 (48.7)
>60 years	60 (55.6)	122 (56.0)*	51 (56.7)	102 (57.0)*	9 (50.0)	20 (51.3)*
Histological subtypes Epithelioid	102 (94.4)	-	84 (93.3)	-	18 (100)	-
Biphasic	2 (1.9)	-	2 (2.2)	-	-	-
Sarcomatoid	4 (3.7)	-	4 (4.5)	-	-	-
Asbestos exposure No	4 (3.7)	19 (8.7)	2 (2.2)	16 (8.9)	2 (11.1)	3 (7.7)
Yes	104 (96.3)	199 (91.3)	88 (97.8)	163 (91.1)**	16 (88.9)	36 (92.3)
Occupational exposure No	26 (24.1)	90 (41.3)	13 (14.4)	57 (31.8)	13 (72.2)	33 (84.6)
Yes	82 (75.9)	128 (58.7)*	77 (85.6)	122 (68.2)*	5 (27.8)	6 (15.4)
Years of occupational exposure [Median (IQR^a^)]	11.5 (2–28)	17.5 (5–27.5)	12 (2–28)	18 (5–29)	10 (8–10)	9.5 (3–24)
Environmental exposure No	31 (28.7)	73 (33.5)	28 (31.1)	60 (33.5)	3 (16.7)	13 (33.3)
Yes	77 (71.3)	145 (66.5)	62 (68.9)	119 (66.5)	15 (83.3)	26 (66.7)
Years of environmental exposure [Median (IQR^a^)]	30 (18–42)	35 (20–46)	29.5 (16–42)	32 (17–49)	31 (30–42)	37.5 (21–42)
Previous chemotherapy No	82 (75.9)	-	69 (76.7)	-	13 (72.2)	-
Yes	26 (24.1)	-	21 (23.3)	-	5 (27.8)	-
Smoking status Non-smoker	39 (36.1)	89 (40.8)	27 (30.0)	63 (35.2)	12 (66.7)	26 (66.7)
Current/ever smoker	69 (63.9)	129 (59.2)	63 (70.0)	116 (64.8)	6 (33.3)	13 (33.3)
Drinking habit Non-drinker	11(10.2)	31 (14.2)	4 (4.4)	11 (6.1)	7 (38.9)	20 (51.3)
Current/ever	97 (89.8)	187 (85.8)	86 (95.6)	168 (93.9)	11 (61.1)	19 (48.7)
Blood pressure ≤120/80 mmHg	83 (76.9)	152 (69.7)	70 (77.8)	126 (70.4)	13 (72.2)	26 (66.7)
>120/80 mmHg	25 (23.1)	66 (30.3)	20 (22.2)	53 (29.6)	5 (27.8)	13 (33.3)
Glucose levels ≤120 mg/dL	77 (79.4)	153 (81.8)	66 (80.5)	129 (82.2)	11 (73.3)	24 (80.0)
>120 mg/dL	20 (20.6)	34 (18.2)	16 (19.5)	28 (17.8)	4 (26.7)	6 (20.0)
Years with diabetes mellitus [Median (IQR)]	10 (3–17)	7 (3–12)	10 (4–16)	7.5 (3–12)	4 (2–20)	6.5 (4–14)
Ureic nitrogen <20 mg/dL	74 (77.9)	163 (86.7)	63 (77.8)	137 (86.7)	11 (78.6)	26 (86.7)
≥20 mg/dL	21 (22.1)	25 (13.3)	18 (22.2)	21 (13.3)	3 (21.4)	4 (13.3)
Creatinine <1.25 mg/dL	92 (94.9)	176 (94.1)	78 (94.0)	146 (93.0)	14 (100)	30 (100)
≥1.25 mg/dL	5 (5.1)	11 (5.9)	5 (6.0)	11 (7.0)	0 (0.0)	0 (0.0)
Total proteins <6 /dL	14 (21.2)	0 (0.0)**	14 (24.6)	0 (0.0)**	0 (0.0)	0 (0.0)
≥6 g/dL	52 (78.8)	184 (100)	43 (75.4)	154 (100)	9 (100)	30 (100)
Total bilirubin <1.45 mg/dL	66 (100)	178 (96.2)	55 (100)	148 (95.5)	11 (100)	30 (100)
≥1.45 mg/dL	0 (0)	7 (3.8)	0 (0.0)	7 (4.5)	0 (0.0)	0 (0.0)
Direct bilirubin ≤0.3 mg/dL	56 (88.9)	176 (95.1)	48 (88.9)	146 (94.2)	8 (88.9)	30 (100)
>0.3 mg/dL	7 (11.1)	9 (4.9)	6 (11.1)	9 (5.8)	1 (11.1)	0 (0.0)
Cholesterol <200 mg/dL	16 (84.2)	95 (50.8)	15 (88.2)	80 (51.0)	1 (50.0)	15 (50.0)
≥200 mg/dL	3 (15.8)	92 (49.2)**	2 (11.8)	77 (49.0)**	1 (50.0)	15 (50.0)
Triglycerides ≤150 mg/dL	28 (82.3)	92 (49.5)	23 (82.1)	83 (52.9)	5 (83.3)	9 (31.0)
>150 mg/dL	6 (17.7)	94 (50.5) *	5 (17.9)	74 (47.1) *	1 (16.7)	20 (69.0)**
Erythrocytes ≤4.5 x10^6^/mm^3^	24 (27.9)	5 (2.7)	22 (30.1)	3 (1.9)	4 (30.8)	2 (6.9)
>4.5 x10^6^/mm^3^	62 (72.1)	182 (97.8)**	51 (69.9)	154 (98.1)**	9 (69.2)	27 (93.1)
Platelets ≤150,000/mm^3^	4 (4.1)	11 (5.9)	3 (3.8)	10 (6.4)	1 (5.9)	1 (3.55)
>150,000/mm^3^	93 (95.9)	175 (94.1)	77 (96.3)	147 (93.6)	16 (94.1)	28 (96.5)
Leukocytes ≤11,000/mm^3^	85 (86.7)	184 (98.4)	70 (85.4)	155 (98.1)	15 (93.7)	29 (100)
>11,000/mm^3^	13 (13.3)	3 (1.6)**	12 (14.8)	3 (1.9)**	1 (6.3)	0 (0.0)

IQR, interquartile range.

*Chi square test (p<0.05).

**Fisher’s exact test (p<0.05).

***Erythrocytes value considered in women was 4.2 x10^6^/mm^3^.

### miR-103a-3p, mesothelin, and calretinin levels

**Fig 1:**
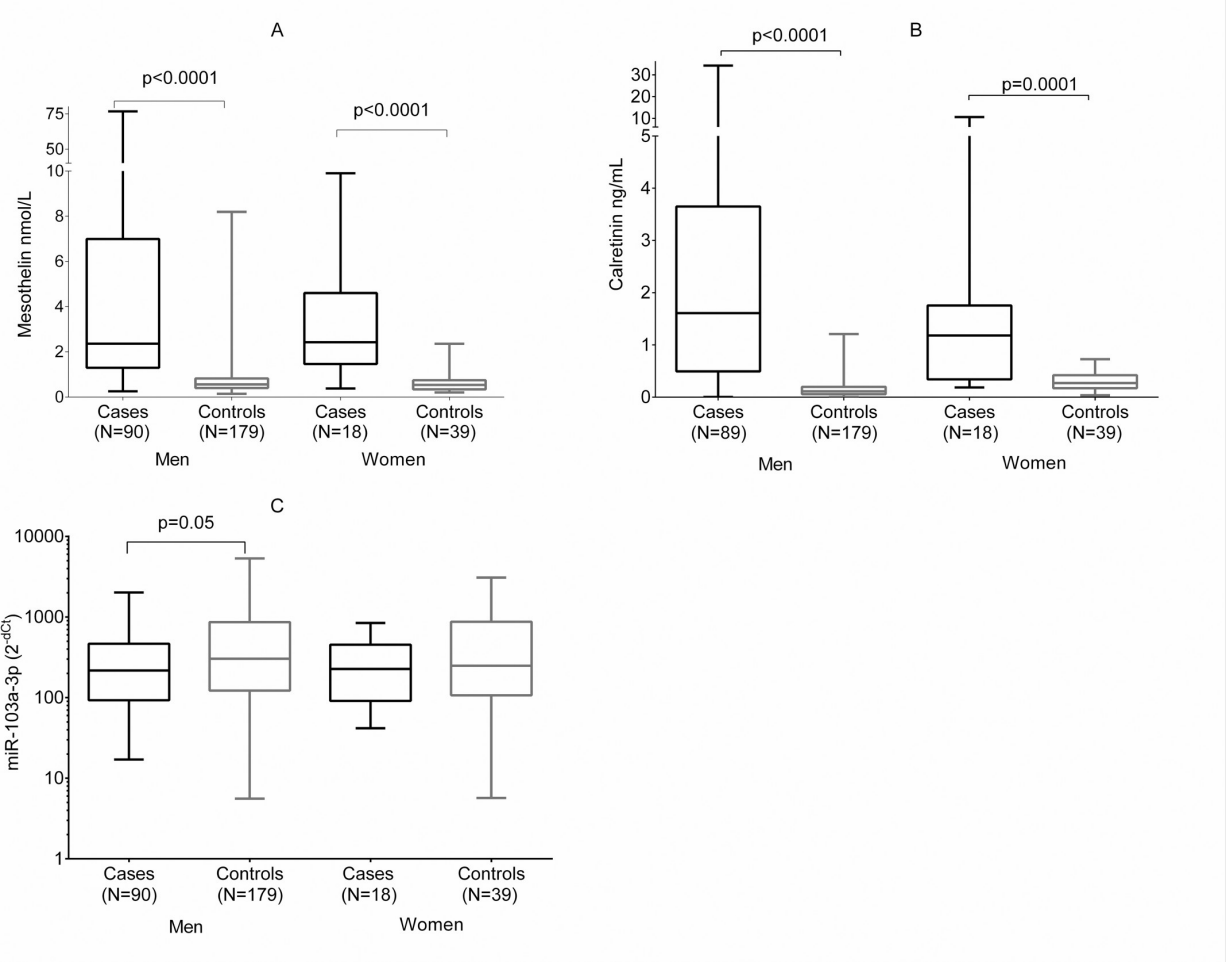
Distribution of medians of mesothelin (A), calretinin (B) and miR-103a-3p (C) in malignant pleural mesothelioma cases and population controls in a Mexican population. Black bars represent cases and gray bars represent controls.

**Fig 2:**
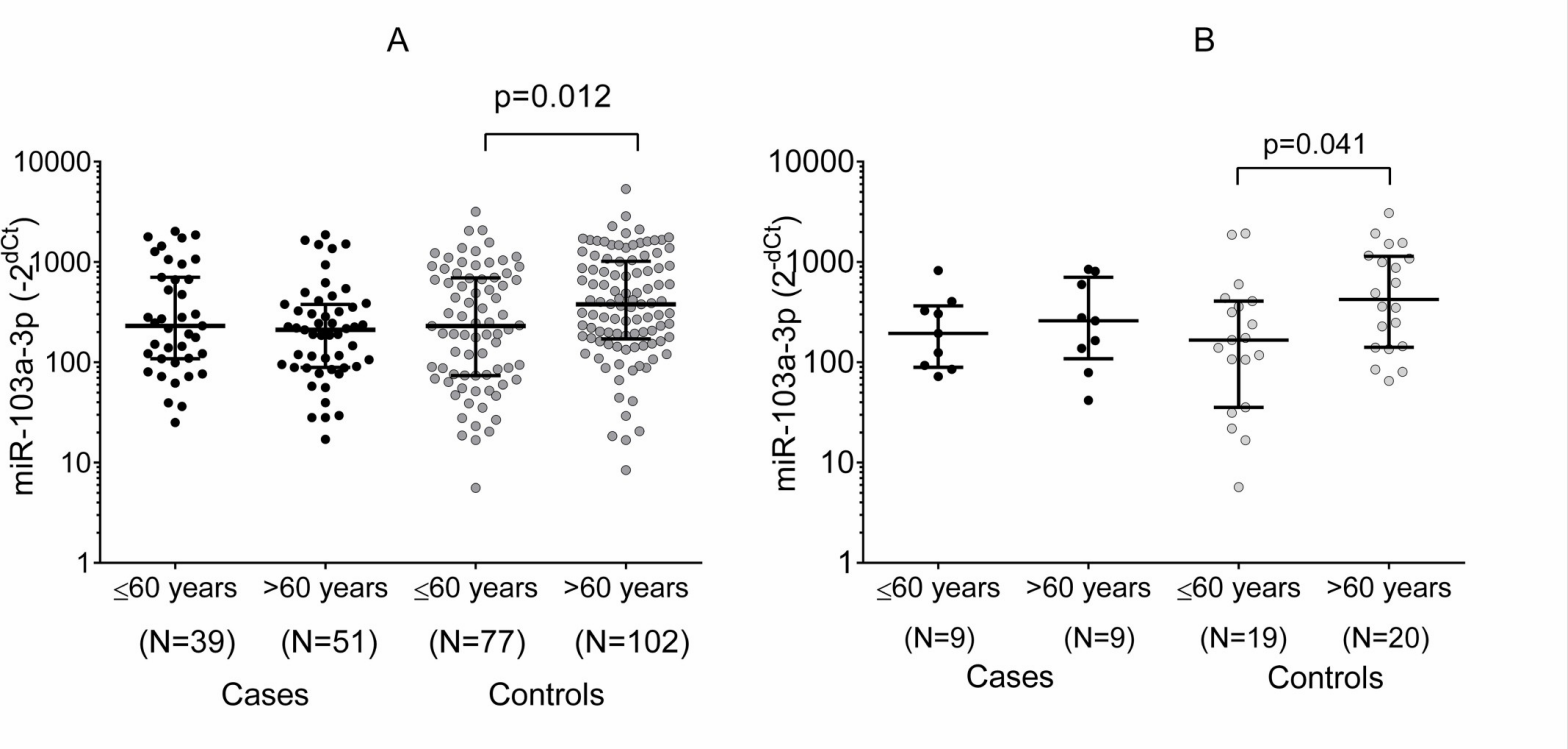
Distribution of miR-103a-3p medians between malignant pleural mesothelioma cases and controls grouped by age and sex [men (A) and women (B)] in a Mexican population. Black dots represent cases, and gray dots controls.

The three biomarkers were determined in a total of 326 samples. The median level of miR-103a-3p in cases was 217.52 and in controls 298.17 (Table 2). Marginally significant differences (p = 0.05) were observed between male cases and controls (217.52 vs. 298.17, respectively) but not within females (Fig 1). Among controls, statistically significant differences were observed between age groups (≤60 years: 203.15 vs. >60 years: 379.03) (Table 2), which remained for men and women (Fig 2).

**Table 2:** Distribution of miR-103a-3p levels in leukocytes among malignant pleural mesothelioma cases and population controls according to sex groups in a Mexican population.

Variables	Total sample		Men		Women	
	Cases Median (IQR)	Controls Median (IQR)	Cases Median (IQR)	Controls Median (IQR)	Cases Median (IQR)	Controls Median (IQR)
mir 103a-3p	**217.52 (94.03–466.33)**	**298.17 (120.25–855.13)** p = 0.036	**217.52 (95.00–474.41)**	**298.17 (121.93–855.13)** p = 0.050	226.79 (93.05–404.50)	248.38 (106.89–872.44)
Subtypes						
Epithelioid	213.80 (90.50–474.41)	-	213.83 (89.57–485.81)	-	226.79 (93.05–404.50)	-
Biphasic	413.10 (121.93–704.27)	-	413.10 (121.93–704.27)	-	-	-
Sarcomatoid	225.55 (162.08–234.92)	-	225.55 (162.08–234.92)	-	-	-
Age						
≤60 years	224.42 (103.71–668.60)	**203.15 (73.77–663.98)**	230.58 (108.38–704.27)	**229.12 (74.02–689.78)**	194.01 (93.05–326.28)	**166.57 (35.50–407.31)**
>60 years	213.80 (89.57–381.71)	**379.03 (162.01–1038.29)** p = 0.001	210.83 (88.64–377.41)	**379.03 (173.64–1009.90)** p = 0.012	259.57 (137.74–596.34)	**423.64 (142.03–1121.23)** p = 0.041
Occupational exposure						
No	*292*.*28 (124*.*49–809*.*00)*	384.13 (140.06–872.44)	304.43 (191.34–1060.11)	418.76 (194.01–868.4)	278.20 (124.49–596.34)	315.17 (117.78–872.44)
Yes	*188*.*06 (90*.*50–386*.*02)*	254.25 (101.26–786.95)	190.01 (90.50–410.14)	262.32 (106.89–797.86)	137.74 (93.05–164.27)	141.55 (65.34–349.70)
	*p = 0*.*082*					
Environmental exposure						
No	219.79 (87.42–410.14)	**202.25 (87.24–666.28)**	202.95 (87.73–366.00)	**196.79 (87.73–678.03)**	809.00 (72.00–843.35)	349.70 (65.34–621.66)
Yes	216.76 (108.38–474.41)	**359.53 (158.16–910.17)** p = 0.024	217.52 (108.38–526.39)	**398.93 (173.64–910.17)** p = 0.027	194.01 (93.05–326.28)	243.61 (135.29–995.99)
Previous Chemotherapy						
No	219.03 (88.64–458.25)	-	218.27 (88.64–458.25)	-	259.57 (124.49–404.50)	**-**
Yes	157.72 (106.15–526.39)	-	151.16 (108.38–526.39)	-	164.27 (93.05–326.28)	**-**
Smoking status						
Non-smoker	177.29 (80.44–386.02)	298.17 (121.93–689.78)	177.29 (80.44–386.02)	298.17 (121.93–689.78)	221.24 (101.64–461.31)	271.35 (117.78–872.44)
Current/ever smoker	219.79 (108.38–474.41)	308.68 (119.42–897.64)	219.79 (108.38–497.22)	308.68 (121.10–903.90)	226.79 (93.05–404.50)	248.38 (106.89–407.31)
Drinking habit						
Non-drinker	278.20 (85.03–809.00)	359.53 (135.29–995.99)	820.35 (275.36–1609.58)	600.49 (76.00–1074.91)	164.27 (78.79–596.34)	332.43 (137.68–934.21)
Current/ever	210.83 (95.00–410.14)	280.13 (119.42–797.86)	201.09 (90.50–458.25)	292.06 (122.36–805.14)	259.57 (124.49–404.50)	227.54 (106.09–621.66)
Blood pressure						
≤120/80 mmHg	194.01 (90.50–404.50)	280.13 (118.60–773.36)	217.52 (90.50–497.22)	284.07 (119.42–770.68)	164.17 (93.05–304.43)	243.61 (117.78–1082.38)
>120/80 mmHg	259.57 (109.89–596.34)	387.70 (135.29–922.88)	217.98 (108.02–434.19)	418.76 (161.60–1009.90)	596.34 (259.57–809.00)	315.17 (106.09–621.66)
Glucose levels						
≤120 mg/dL	218.27 (88.64–377.41)	393.44 (166.57–995.99)	222.88 (88.64–526.39)	393.44 (162.01–922.88)	164.27 (85.03–278.20)	383.42 (170.71–1121.2)
>120 mg/dL	252.63 (104.47–708.74)	399.70 (188.70–739.29)	205.23 (104.47–398.08)	472.58 (198.92–775.85)	708.74 (344.69–832.25)	137.20 (31.34–621.666)
Ureic nitrogen						
<20 mg/dL	206.14 (90.50–386.02)	**436.54 (177.29–1038.29)**	218.27 (90.50–474.41)	**486.86 (188.70–989.11)**	194.01 (85.03–326.28)	383.42 (140.06–1160.07)
≥20 mg/dL	280.13 (185.33–704.27)	**221.07 (106.89–380.65)** p = 0.016	299.45 (185.33–704.27)	**221.07 (94.35–380.65)** p = 0.020	259.57 (93.05–843.35)	242.50 (121.09–672.85)
Creatinine						
<1.25 mg/dL	214.55 (94.03–442.28)	396.18 (170.10–995.99)	219.03 (95.00–497.22)	403.12 (184.82–989.11)	179.14 (93.05–304.43)	359.53 (139.10–1082.38)
≥1.25 mg/dL	280.13 (109.89–324.43)	229.12 (133.43–486.86)	280.13 (109.89–324.43)	229.12 (133.43–486.86)	-	-
Total proteins						
<6 g/dL	215.31 (88.64–301.99)	-	215.31 (88.64–301.99)	-	-	**-**
≥6 g/dL	279.17 (141.87–746.03)	390.52 (164.29–955.99)	280.13 (146.01–955.42)	396.18 (177.29–916.07)	259.57 (137.74–304.43)	359.53 (139.10–1082.38)
Total bilirubin						
<1.5 mg/dL	245.57 (109.89–666.28)	390.52 (166.57–922.88)	247.27 (109.89–704.27)	396.18 (181.05–913.12)	194.01 (93.05–304.43)	359.53 (139.10–1082.38)
≥1.5 mg/dL	-	242.19 (87.24–1038.29)	-	242.19 (87.24–1038.29)	-	-
Direct bilirubin						
<0.3 mg/dL	265.08 (128.58–685.28)	**403.12 (170.10–995.99)**	275.36 (132.72–820.02)	**411.59 (184.82–989.11)**	226.79 (108.26–291.32)	359.53 (139.10–1082.38)
≥0.3 mg/dL	190.01 (88.03–670.92)	**187.40 (87.24–364.55)**	149.95 (88.03–670.92)	**187.40 (87.24–364.55)**	596.34	-
		p = 0.036		p = 0.032		
Cholesterol						
<200 mg/dL	320.30 (173.65–668.60)	418.76 (191.34–872.44)	354.58 (280.13–670.92)	413.04 (191.34–846.33)	41.64	436.54 (166.57–1082.38)
≥200 mg/dL	191.34 (39.67–821.15)	357.06 (131.20–1024.09)	115.50 (39.67–191.34) p = 0.073	377.41 (151.16–1009.90)	821.15	349.70 (84.44–1160.07)
Triglycerides						
≤150 mg/dL	245.57 (131.11–453.68)	370.98 (188.74–891.30)	247.27 (146.01–410.14)	380.65 (192.67–922.88)	137.74 (124.49–596.34)	248.38 (139.10–436.54)
>150 mg/dL	154.03 (39.67–194.01)	403.12 (146.01–1038.29)	116.73 (39.67–191.34)	403.12 (151.16–916.07)	191.01	423.64 (137.68–11340.11)
Erythrocytes						
≤4.5x10^6^/ mm^3^	**282.87 (194.01–526.39)**	162.01 (87.24–359.53)	**293.80 (225.97–526.39)**	162.01 (87.24–296.11)	179.14 (102.96–507.58)	249.80 (112.25–1721.14)
>4.5x10^6^/ mm^3^	**190.01 (88.34–390.95)** p = 0.035	407.31 (173.64–995.99)	**151.16 (84.44–377.41)** p = 0.007	411.59 (177.29–922.88)	304.43 (259.57–404.50)	407.31 (166.57–1082.38))
Platelets						
≤150000/ mm^3^	544.57 (235.07–1227.55)	491.14 (184.82–2105.57)	280.13 (190.01–1646.10)	434.27 (184.82–1715.60)	809.00	3082.74
>150000/ mm^3^	218.27 (99.04–410.14)	380.65 (161.60–922.88)	218.27 (97904–474.41)	387.61 (162.01–916.07)	226.79 (108.77–365.39)	359.53 (139.58–1039.19)
Leukocytes						
≤11000/mm^3^	210.83 (90.50–497.22)	393.27 (170.10–992.55)	213.80 (88.64–526.39)	398.93 (177.29–922.88)	194.01 (93.05–404.50)	359.53 (140.06–1082.38)
>11000/mm^3^	218.27 (185.33–280.13)	119.42 (8.39–812.42)	204.80 (146.85–299.45)	119.42 (8.39–812.42)	259.57	-

IQR, Interquartile range.

All comparisons were tested with Mann-Whitney U test.

Statistically significant differences were also found among male controls with environmental exposure to asbestos (without exposure: 202.25 vs. with exposure: 359.53), urea nitrogen levels and direct bilirubin (Table 2). Also, statistically significant differences were observed in male cases according to different erythrocytes levels (≤4.5 x10^6^/mm^3^: 293.80 vs >4.5 x 10^6^/mm^3^: 151.16). Moreover, miRNAs did not differ by drinking habits (non-drinker: 820.35 vs. drinker: 201.09; p = 0.053) and by blood pressure levels in controls (280.13 vs. 387.70 between blood pressure below/equal to and above 120/80 mmHg, respectively; p = 0.286). Finally, among female cases differences were found by glucose levels below and above 120 mg/dL (164.27 vs. 708.74) still not statistically significant (Table 2).

Median mesothelin levels were higher in cases compared to controls (2.34 and 0.55 nmol/L; S1 Table), which remained statistically significant after sex stratification–male cases 2.34 nmol/L and controls 0.56 nmol/L; female cases 2.28 nmol/L and controls: 0.53 nmol/L (Fig 1). Likewise, male controls >60 years presented higher mesothelin levels compared to those ≤60 years (0.62 vs. 0.48 nmol/L; p = 0.001) (S1 Table). Respecting calretinin, cases presented significantly higher levels compared to controls (1.52 vs. 0.13 ng/mL), which remained between males and females (Fig 1). Noteworthy, calretinin levels were significantly higher between men and women only within controls (0.11 ng/mL vs. 0.27 ng/mL) (S1 Table).

### miR-103a-3p correlations with different variables

**Fig 3:**
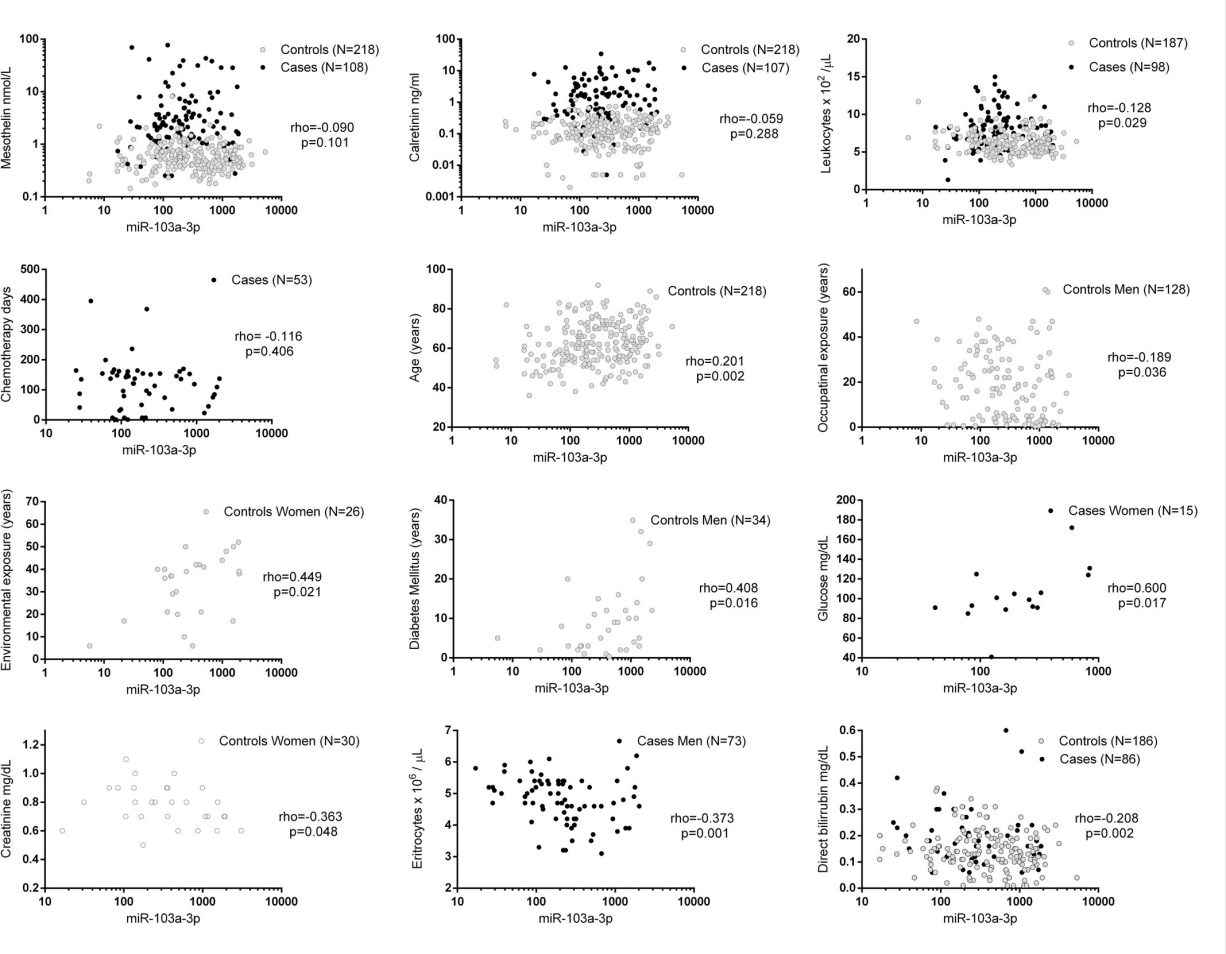
Correlation of miR-103a-3p with different variables in malignant pleural mesothelioma cases and population controls in a Mexican population.

Mir-103a-3p levels showed no linear correlation with mesothelin and calretinin concentrations (Fig 3). MiR-103a-3p was negatively correlated with age in male cases (Spearman -0.08; 95% CI -0.29–0.13) and positively correlated in male controls (Spearman 0.18; 95% CI 0.04–0.32). Likewise, positive correlations with environmental asbestos exposure were observed among females. Similarly, the correlations with diabetes mellitus duration among male controls and blood glucose levels among female controls were moderately to strongly positive (Fig 3) (S2 Table).

On the other hand, years of occupational exposure presented a negative correlation within male controls, yet the correlation was weak (ρ = -0.128, p = 0.029). Moreover, leukocyte levels, creatinine levels among female controls, direct bilirubin levels and erythrocytes among males were negatively correlated with miR-103a-3p (Fig 3) (S2 Table).

### Individual and combined sensitivity and specificity of miR-103a-3p, mesothelin, and calretinin

**Fig 4:**
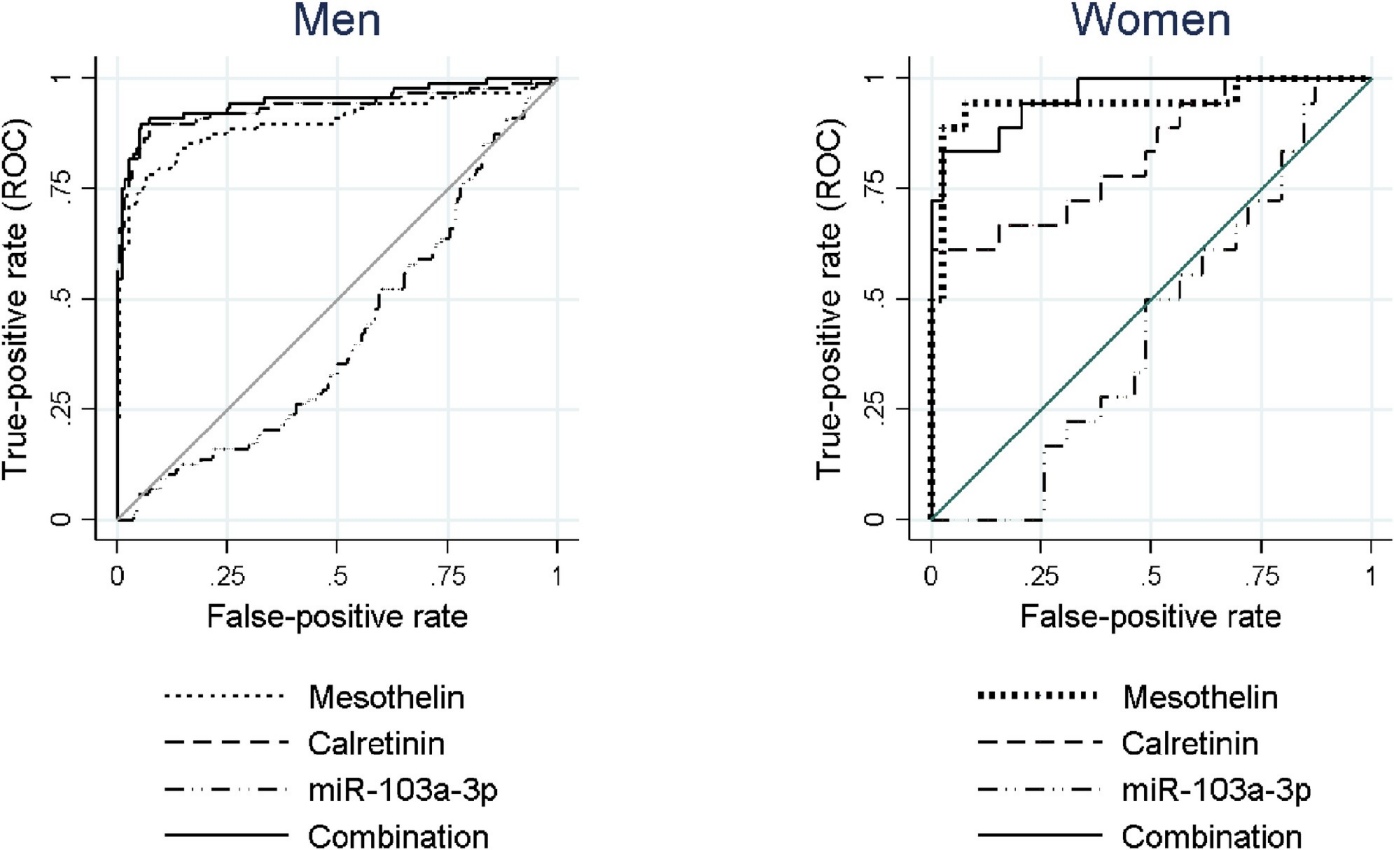
ROC curves for mesothelin, calretinin and miR-103a-3p in men and women. Areas under the curve are shown for each biomarker, individually and in combination.

At a fixed specificity of 95.5% for men and 97.4% for women, miR-103a-3p in both males and females presented low AUCs [0.426 (95% CI 0.355–0.497) and 0.437 (95% CI 0.284–0.589), respectively], with a sensitivity of 4.4% in men and 0% in women. Among males, an AUC of 0.894 (95% CI 0.847–0.941) and a sensitivity of 72.2% at 95.5% specificity was reported for mesothelin, whereas for calretinin an AUC of 0.931 (95% CI 0.889–0.972) and a sensitivity of 80.9% at 95.5% specificity was observed. Regarding females, mesothelin had a better performance, with an AUC of 0.947 (95% CI: 0.870–1.024) and a sensitivity of 88.9% at 97.4% specificity, in contrast to calretinin [AUC = 0.829 (95% CI: 0.706–0.951), 61.1% sensitivity and 97.4% specificity]. When mesothelin and calretinin were combined, sensitivity reached 80.9% in men (95.5% specificity) and 83.3% in women (97.4% specificity). When miR-103a-3p was included together with both biomarkers, there was no increase in sensitivity (Fig 4 and Table 3). Regarding age-related differences of miR-103a-3p levels, additional analyses were conducted in males >60 years (51 cases and 102 controls) and ≤60 years (39 cases and 77 controls), revealing improved performance of miR-103a-3p when stratified by age in terms of a larger area under the curve in the group of older men (0.6584 vs. 0.5361)–among participants >60 years miR-103a-3p cutoff ≤39.671 resulted in a sensitivity of 9.8% and a specificity of 95.1%, whereas among participants aged ≤60 years, with miR-103a-3p cutoff >1438.152 resulted in a sensitivity of 12.8% and a specificity of 94.8% (S3 Table). Despite a doubling of the sensitivity of miR-103-3p as an individual marker in the subpopulation, the addition of miR-103a-3p to the marker combination of mesothelin and calretinin did not lead to an improved performance.

**Table 3:** Sensitivity and specificity of biomarkers among malignant pleural mesothelioma cases and population controls according to sex groups in a Mexican population.

Biomarkers	Men							
	Cut-off	AUC (95% CI)	Sensitivity (%)	Specificity (%)	TP (N)	TN (N)	FP (N)	FN (N)
Mesothelin	1.379 nmol/L	0.894 (0.847–0.941)	72.2	95.5	62	174	5	28
Calretinin	0.369 ng/ml	0.931 (0.889–0.972)	80.9	95.5	70	173	6	19
miR-103a-3p	1782	0.426 (0.355–0.497)	4.4	95.5	4	171	8	86
Mesothelin, calretinin, miR-103a-3p		0.945 (0.910–0.979)	80.9	95.5	72	174	5	17
Mesothelin and calretinin		0.943 (0.909–0.977)	80.9	95.5	71	174	5	18
Mesothelin and miR-103a-3p		0.894 (0.848–0.941)	72.2	95.5	62	174	5	28
Calretinin and miR-103a-3p		0.932 (0.893–0.971)	80.9	95.5	70	173	6	19
**Biomarkers**	**Women**							
	**Cut-off**	**AUC (95% CI)**	**Sensitivity** **(%)**	**Specificity** **(%)**	**TP (N)**	**TN (N)**	**FP (N)**	**FN (N)**
Mesothelin	1.275 nmol/L	0.947 (0.870–1.024)	88.9	97.4	16	38	1	2
Calretinin	0.726 ng/ml	0.829 (0.706–0.951)	61.1	97.4	11	38	1	7
miR-103a-3p	3082	0.437 (0.284–0.589)	0	97.4	0	39	0	18
Mesothelin, calretinin, miR-103a-3p		0.958 (0.907–1.000)	83.3	97.4	15	38	1	3
Mesothelin and calretinin		0.951 (0.886–1.016)	83.3	97.4	15	38	1	3
Mesothelin and miR-103a-3p		0.954 (0.893–1.014)	72.2	97.4	14	38	1	4
Calretinin and miR-103a-3p		0.864 (0.758–0.970)	55.6	97.4	11	36	3	7

TP, true positives; TN, true negatives; FP, false positives; FN, false negatives; AUC, area under the curve; CI, confidence interval.

AUC, cut-offs, and sensitivity of individual biomarkers were calculated with ROC curves, at a specificity of 95%.

## Discussion

Several studies in different populations have analyzed the combination of certain biomarkers at different molecular levels to improve MPM diagnosis [6–8,20,25,29]. In this regard, our study aimed to assess miR-103a-3p in leukocytes in addition to mesothelin and calretinin in plasma. However, the addition of this third miRNA marker did not increase the performance of mesothelin and calretinin for MPM diagnosis in a Mexican population.

Initially, miR-103a-3p was described as a biomarker for mesothelioma using the cellular fraction of blood [19]. A further study by Weber et al. confirmed that miR-103a-3-p was lower in individuals with MPM in comparison to asbestos-exposed controls and controls from the general population, but there were no differences according to histological subtypes [25]. In our study miR-103-3p was also detectable in leukocytes and lower levels were observed in MPM cases compared to controls. However, differences were present only among males, whereas among females no differences in miR-103-3p were observed between cases and controls. In contrast, Weber et al. reported no differences in miR-103a-3p levels between men and women, regardless of case and control status, probably due to small sample size–five female cases and one asbestos-exposed control [19]. Hence, more research efforts in bigger study groups are needed to evaluate the expression of miR-103a-3p in women. Regarding smoking status, our results for miR-103a-3p were consistent with those reported by Weber et al. with no differences between smokers and non-smokers [19]. We also observed a higher expression of miR-103a-3p among controls aged >60 years, which could be explained by the presence of non-communicable diseases such as high blood pressure, diabetes mellitus, osteoporosis, arthritis, decreased kidney function, and cardiovascular diseases, which cause certain miRNAs to be over- or under-expressed [30]. Nonetheless, this overexpression among males >60 years did not improve the diagnostic performance of the combination mesothelin plus calretinin. Particularly, high miR-103a-3p plasma levels have been reported among individuals with high blood pressure and kidney injury [31,32]. This might be relevant because reduced renal function can be an influencing factor of circulating biomarkers as has been shown for calretinin and mesothelin [33]. In our study, only one individual presented with chronic kidney disease and unfortunately no markers of early renal damage were determined to evaluate the behavior of miR-103a-3p in this clinical condition. In the case of high blood pressure, we did not find differences in miR-103a-3p expression, possibly because this information was obtained by questionnaire and not by measurement. The differences found in male controls in relation to urea nitrogen levels in our study could support this issue. However, in order to clarify the mechanisms behind kidney function and miRNA expression, it is necessary to evaluate miR-103a-3p in different metabolic conditions. For instance, in our study these conditions were only evaluated by questionnaire or determined indirectly by biochemical parameters such as glucose, blood ureic nitrogen, and creatinine. Likewise, it was not possible to compare our results by histological subtype, since the number of cases with biphasic and sarcomatoid subtypes was too small.

Although miR-103a-3p has been reported to be upregulated in newly diagnosed diabetes mellitus (less than 5 years old), high blood pressure and kidney damage [31,32,34], in our study no significant changes in the expression of this miRNA were observed in relation to glucose levels >120 mg/dL. However, there were positive correlations in male controls with respect to years of diabetes mellitus and with glucose levels in female cases. It was previously reported that the miR-103a family could function as a biomarker of diabetes [35]. Considering that the prevalence of diabetes mellitus in Mexico in the population aged 60–69 years is 25.8%, and that in 2020 it was ranked third among all causes of mortality, its application could be explored as a marker for early diagnosis or surveillance in diabetic people in the Mexican population [36]. The negative correlation of miR-103a-3p with creatinine levels in the control group of women could suggest an involvement in kidney damage, as previously reported by Lu et al [37]. Remarkably, a positive correlation was found with environmental exposure for female controls but negative for males with occupational asbestos exposure. This might suggest that miR-103a-3p could possibly be useful as a marker of asbestos exposure rather than an MPM hallmark. Another finding in our study was that miR-103a-3p was positively correlated with age and was significantly different between participants >60 years and ≤60 years within the control group, similar to previously reported results [38].

Although it has been reported that miR-103a-3p can discriminate between MPM patients and people exposed to asbestos using extracellular plasma vesicles, when exploring its use as a prognostic biomarker in patients with MPM and after chemotherapy in the same biological matrix, the results have been conflicting [39,40]. Therefore, there is a need for further research to clarify the role of miR-103a-3p as a prognostic biomarker in patients with MPM. On the other hand, in other types of cancer such as colon cancer, breast cancer, and prostate cancer, miR-103a-3p has been considered a good candidate biomarker for diagnostics. In case of breast cancer, miR-103a-3p has shown to be upregulated in patients with breast tumors and after surgery the expression levels of this miRNA decreases, suggesting a potential role as a marker for treatment follow-up [41–43]. Within some other diseases such as endometriosis and fibromyalgia, it has also been considered as a promising non-invasive diagnostic candidate [44,45].

An analysis of a possible correlation of miR-103a-3p with other miRNAs that have shown diagnostic potential with MPM was not part of the current study but might be of interest in this context. We and others have previously tested miR-126 and miR-132-3p with MPM cases and controls [20,29]. Using data from a recent publication [46] we could not see a positive correlation between miR-103a-3p and either miR-126 or miR-132-3p.

For mesothelin and calretinin, we found significant differences in cases and controls, which remained after stratified analysis by sex and age. In the case of calretinin, significant differences were observed within controls as expected, since most of the samples corresponded to a subsample of the study by Jiménez-Ramírez et al. [6].

Weber et al. reported that the combination of miR-103a-3p with mesothelin increased the sensitivity for MPM diagnosis up to 81% with 95% specificity, but this could not be shown in this Mexican study group. However, the previous study group was older (median age 72 years in cases and 73 years in controls) in contrast to 62 years in cases and controls in this study group, for which is also an association of miR-103a-3p with age is shown. For Mexican men >60 years an improvement of the sensitivity of miR-103a-3p could be observed. Thus, in future studies it is indicated to analyze the association of miR-103a-3p with age in more detail. Generally, further research involving a different population with a larger sample size is needed, including more female participants [25]. With respect to our results, low AUC and sensitivity for miR-103a-3p were found. In addition, miR-103a-3p in leukocytes could not differentiate between MPM cases and healthy participants, possibly due to the analyzed matrix, i.e., isolated leukocytes instead of the whole cellular fraction of blood, and the corresponding different isolation procedures. As Podolska et al. previously reported, miRNAs were sensitive to the used isolation procedure [47]. Also, the inclusion of miR-103a-3p in any combination with mesothelin or calretinin did not substantially improve sensitivity, despite our larger study group compared to the study by Weber et al. [25]. These differences could be determined by ethnicity, along with the variability in a specific miRNA within the same population, which might hinder miRNAs’ use as a potential biomarker [48].

In the present study the combination of mesothelin and calretinin showed good sensitivities for both males and females (80.9 and 83.3, respectively). By including miR-103a-3p, the sensitivity for MPM diagnosis was not improved, because its performance as an individual marker was already negligible. In addition, the performance of mesothelin and calretinin was clearly better in the Mexican population compared to the German study group used by Weber et al., with no improvement by adding a third biomarker. It is likely that miR-103a-3p has greater utility in the Mexican population as an indicator of metabolic conditions rather than as a diagnostic biomarker of MPM. However, it would be important to evaluate this miRNA in a larger number of women in order to assess the correlation with other characteristics, such as weight, height, kidney function, glycosylated hemoglobin, etc. Finally, future studies should consider screening for miRNAs in the Mexican population to determine which miRNAs are deregulated, in order to evaluate these candidate biomarkers for MPM diagnosis.

In conclusion, the addition of miR-103a-3p to the established biomarker panel comprising of mesothelin and calretinin did not improve the diagnostic performance for MPM diagnosis. Still, miR-103a-3p levels were correlated with several characteristics not yet explored in a Mexican population, which could be useful for other purposes rather than diagnostics. Further research should aim to explore the potential clinical use of miR-103a-3p for diagnostic and prognostic purposes including chronic diseases or aberrant biochemical parameters in the Mexican population.

## Supporting information

S1 Fig Comparison of batches. Differences between cases and controls of 103a-3p (2^-dCt^) measurements performed in different years of the samples included in this study (A) and differences between miR-103a-3p (2^-dCt^) measurements performed in different years of all samples included in this study (B).
(DOCX)

S1 Table Distribution of mesothelin and calretinin concentrations in a study of MPM cases and control populations in a Mexican population. (DOCX)

S2 Table Correlations between miR-103a-3p and hematological and biochemical parameters and years of asbestos exposure in MPM cases and population controls in a Mexican population. (DOCX)

S3 Table Biomarker performance in the group of Mexican men stratified by age. (DOCX)

S1 Dataset Dataset of biomarker values and basic clinical parameters. (XLSX)
