# Supplementary material for: Assessment of miR-103a-3p in leukocytes—No diagnostic benefit in combination with the blood-based biomarkers mesothelin and calretinin for malignant pleural mesothelioma diagnosis
Source: PLoS One. 2022 Oct 14;17(10):e0275936. doi: 10.1371/journal.pone.0275936 (PMC9565669; doi:10.1371/journal.pone.0275936)
Supplement: S1 Fig — Differences between cases and controls of 103a-3p (2-dCt) measurements performed in different years of the samples included in this study (A) and differences between miR-103a-3p (2-dCt) measurements performed in different years of all samples included in this study (B). (DOCX) [file pone.0275936.s001.docx]

**A**

**B**

**S1 Figure. Comparison of batches.** Differences between cases and controls of 103a-3p (2^-dCt^) measurements performed in different years of the samples included in this study (A) and differences between miR-103a-3p (2^-dCt^) measurements performed in different years of all samples included in this study (B).
