# Supplementary material for: Assessment of miR-103a-3p in leukocytes—No diagnostic benefit in combination with the blood-based biomarkers mesothelin and calretinin for malignant pleural mesothelioma diagnosis
Source: PLoS One. 2022 Oct 14;17(10):e0275936. doi: 10.1371/journal.pone.0275936 (PMC9565669; doi:10.1371/journal.pone.0275936)
Supplement: S1 Table — (DOCX) [file pone.0275936.s002.docx]

**S1 Table.** **Distribution of mesothelin and calretinin concentrations in a study of malignant pleural mesothelioma cases and control populations in a Mexican population.**

| Variables | Total sample | | Men | | Women | |
| --- | --- | --- | --- | --- | --- | --- |
|  | Cases  Median (IQR) | Controls  Median (IQR) | Cases  Median (IQR) | Controls  Median (IQR) | Cases  Median (IQR) | Controls  Median (IQR) |
| **Mesothelin (nmol/L)** | **2.34** **(1.33-5.18)*** | **0.55** **(0.40-0.78)*** | **2.34 (1.28-6.63)*** | **0.56** **(0.40-0.82)***  p<0.0001 | **2.28** **(1.46-3.79)*** | **0.53** **(0.33-0.74)*** |
| Subtypes (N) |  |  |  |  |  |  |
| Epithelioid (102) | 2.31 (1.31-6.19) | - | 2.31 (1.27-7.54) | - | 2.28 (1.46-3.79) | - |
| Biphasic (2) | 2.97 (2.18-3.76) | - | 2.97 (2.18-3.76) | - | - | - |
| Sarcomatoid (4) | 3.04 (1.58-4.17) | - | 3.04 (1.58-4.17) | - | - | - |
|  |  |  |  |  |  |  |
| Age  **≤**60 years (144) | 2.16 (1.29-5.18) | **0.48 (0.34-0.70)** | 2.18 (1.27-7.92) | **0.48 (0.34-0.70)** | 1.61 (1.35-3.23) | 0.51 (0.31-0.67) |
| >60 years (182) | 2.60 (1.38-5.53) | **0.62 (0.44-0.84)*** | 2.49 (1.28-6.57) | **0.62 (0.44-0.85)*** | 2.71 (2.05-4.60) | 0.58 (0.42-0.79) |
| Smoking status |  |  |  |  |  |  |
| Non-smoker (128) | 2.71 (1.46-4.60) | 0.53 (0.39-0.75) | 2.62 (1.31-4.93) | 0.51 (0.39-0.75) | 2.97 (1.96-4.19) | 0.55 (0.37-0.76) |
| Current/ever smoker (198) | 2.13 (1.27-6.57) | 0.58 (0.40-0.82) | 2.28 (1.27-7.34) | 0.58 (0.41-0.85) | 1.53 (1.27-2.13) | 0.51 (0.33-0.61) |
|  |  |  |  |  |  |  |
| **Calretinin (ng/mL)** | **1.52 (0.44-3.36)** | **0.13 (0.06-0.22)*** | **1.60 (0.48-3.44)** | **0.11 (0.05-0.19)*** | **1.22 (0.34-1.75)** | **0.27 (0.17-0.42)*** |
| Subtypes (N) |  |  |  |  |  |  |
| Epithelioid (101) | 1.52 (0.45-3.36) | - | 1.61 (0.51-3.94) | - | 1.22 (0.34-1.75) | - |
| Biphasic (2) | 2.52 (1.60-3.44) | - | 2.52 (1.60-3.44) | - | - | - |
| Sarcomatoid (4) | 0.38 (0.27-1.20) | - | 0.38 (0.27-1.20) | - | - | - |
|  |  |  |  |  |  |  |
| Age **≤**60 years (144) | 1.50 (0.45-3.40) | 0.13 (0.06-0.22) | 1.52 (0.48-5.36) | 0.10 (0.05-0.20) | 0.8  6 (0.34-1.59) | 0.29 (0.16-0.42) |
| >60 years (181) | 1.64 (0.42-3.37) | 0.13 (0.07-0.21) | 1.62 (0.44-3.24) | 0.11 (0.06-0.18) | 1.73 (0.38-5.08) | 0.26 (0.17-0.37) |
| Smoking status |  |  |  |  |  |  |
| Non-smoker (127) | 0.86 (0.41-2.27) | 0.13 (0.06-0.25) | 0.83 (0.44-2.72) | 0.10 (0.04-0.20) | 1.31 (0.36-2.01) | 0.23 (0.17-0.42) |
| Current/ever smoker (198) | 1.61 (0.48-3.72) | 0.12 (0.07-0.20) | 1.66 (0.48-4.35) | 0.11 (0.06-0.18) | 1.18 (0.22-1.59) | 0.29 (0.17-0.35) |
|  |  |  |  |  |  |  |

*Mann-Whitney U test (p<0.05) ^a^Interquartile range
