# Supplementary material for: Assessment of miR-103a-3p in leukocytes—No diagnostic benefit in combination with the blood-based biomarkers mesothelin and calretinin for malignant pleural mesothelioma diagnosis
Source: PLoS One. 2022 Oct 14;17(10):e0275936. doi: 10.1371/journal.pone.0275936 (PMC9565669; doi:10.1371/journal.pone.0275936)
Supplement: S2 Table — (DOCX) [file pone.0275936.s003.docx]

**S2 Table.** **Correlations between miR-103a-3p, hematological, biochemical parameters and years of asbestos exposure in malignant pleural mesothelioma cases and population controls in a Mexican population.**

| Variable | Total sample  (rho) | | | Men  (rho) | | | Women  (rho) | | |
| --- | --- | --- | --- | --- | --- | --- | --- | --- | --- |
|  | Total | Cases | Controls | Total | Cases | Controls | Total | Cases | Controls |
| Mesothelin | -0.090 | 0.062 | -0.029 | -0.079 | 0.014 | 0.021 | -0.137 | 0.426 | -0.257 |
| Calretinin | -0.059 | 0.094 | 0.018 | -0.064 | 0.071 | 0.031 | 0.058 | 0.164 | 0.090 |
| Age | **0.135*** | -0.022 | **0.201*** | 0.102 | -0.083 | **0.181*** | **0.273*** | 0.278 | 0.257 |
| Years of occupational exposure | **-0.118*** | -0.053 | -0.168 | -0.132 | -0.051 | **-0.189*** | 0.100 | -0.205 | 0.142 |
| Years of environmental exposure | **0.148*** | 0.104 | **0.159*** | **0.152*** | 0.192 | 0.134 | 0.232 | -0.231 | **0.449*** |
| Chemotherapy days | - | -0.116 | - | - | -0.174 | - | - | -0.273 | - |
| Years with Diabetes mellitus | 0.141 | 0.198 | 0.200 | 0.212 | 0.061 | **0.408*** | -0.053 | 0.200 | -0.231 |
| Leukocyte count | **-0.128*** | 0.082 | -0.099 | -0.081 | 0.102 | -0.042 | **-0.378*** | -0.039 | **-0.398*** |
| Erythrocytes | 0.018 | **-0.337*** | 0.052 | 0.025 | **-0.373*** | 0.064 | -0.052 | 0.019 | -0.103 |
| Platelets | -0.096 | 0.146 | -0.045 | -0.065 | 0.146 | -0.002 | -0.245 | 0.183 | -0.293 |
| Absolute lymphocytes | -0.041 | -0.034 | -0.103 | -0.008 | -0.032 | -0.061 | -0.221 | -0.064 | -0.340 |
| Absolute monocytes | -0.060 | 0.147 | -0.030 | -0.049 | 0.196 | -0.038 | -0.143 | -0.239 | -0.012 |
| Absolute neutrophils | **-0.151*** | 0.034 | -0.098 | -0.104 | 0.029 | -0.024 | -**0.414*** | 0.085 | **-0.455*** |
| Absolute eosinophils | -0.015 | 0.087 | -0.087 | -0.041 | 0.074 | -0.121 | 0.149 | 0.378 | 0.068 |
| Absolute basophils | 0.006 | 0.093 | -0.071 | -0.002 | 0.081 | -0.080 | 0.039 | 0.115 | -0.082 |
| Glucose levels | 0.065 | 0.044 | 0.062 | 0.060 | -0.030 | 0.093 | 0.075 | **0.600*** | -0.114 |
| Urea nitrogen | -0.036 | 0.033 | -0.106 | -0.029 | 0.057 | -0.115 | -0.074 | -0.112 | -0.084 |
| Creatinine | 0.063 | 0.082 | -0.004 | 0.091 | 0.055 | 0.047 | -0.211 | 0.197 | **-0.363*** |
| Total proteins | 0.012 | -0.028 | -0.007 | 0.021 | 0.010 | -0.008 | -0.017 | -0.225 | 0.027 |
| Albumin | 0.080 | -0.163 | 0.057 | 0.073 | -0.148 | 0.067 | 0.093 | 0.071 | -0.049 |
| Total bilirubin | -0.018 | -0.153 | -0.002 | -0.055 | -0.242 | -0.021 | 0.025 | 0.345 | -0.055 |
| Direct bilirubin | **-0.173*** | -0.199 | -0.140 | **-0.208*** | **-0.292*** | **-0.165*** | -0.030 | 0.226 | -0.031 |
| Cholesterol | -0.077 | 0.033 | -0.098 | -0.080 | 0.012 | -0.095 | -0.050 | - | -0.014 |
| Triglycerides | -0.023 | -0.212 | -0.046 | -0.061 | -0.236 | -0.081 | 0.186 | 0.028 | 0.123 |

*****Spearman Correlation p<0.05 rho=Spearman coefficient

Correlation analysis was not performed with cholesterol data in female cases because data from only two participants were available.
