# Supplementary material for: Assessment of miR-103a-3p in leukocytes—No diagnostic benefit in combination with the blood-based biomarkers mesothelin and calretinin for malignant pleural mesothelioma diagnosis
Source: PLoS One. 2022 Oct 14;17(10):e0275936. doi: 10.1371/journal.pone.0275936 (PMC9565669; doi:10.1371/journal.pone.0275936)
Supplement: S3 Table — (DOCX) [file pone.0275936.s004.docx]

**S3 Table. Biomarker performance in the group of Mexican men stratified by age**

|  | Cut-off | AUC (95% CI) | Sens (%) | Spec (%) | TP (N) | TN (N) | FP (N) | FN (N) |
| --- | --- | --- | --- | --- | --- | --- | --- | --- |
|  | Men > 60 years of age | | | | | | | |
| Mesothelin [nmol/L] | 1.465 | 0.8910 (0.8277-0.9543 | 70.6 | 95.1 | 36 | 97 | 5 | 15 |
| Calretinin [ng/ml] | 0.309 | 0.9247 (0.8635-0.9859) | 80.0 | 95.1 | 40 | 97 | 5 | 10 |
| miR-103a-3p (normalized) | 39.671 | 0.6584 (0.5678-0.7490) | 9.8 | 95.1 | 5 | 97 | 5 | 46 |
| Mesothelin, calretinin, miR-103a-3p | - | 0.9347 (0.8817-0.9877) | 88.0 | 95.1 | 44 | 97 | 5 | 6 |
| Mesothelin and calretinin | - | 0.9300 (0.8706-0.9894) | 88.0 | 95.1 | 44 | 97 | 5 | 6 |
| Mesothelin and miR-103a-3p | - | 0.8923 (0.8297-0.9550) | 68.6 | 95.1 | 35 | 97 | 5 | 16 |
| Calretinin and miR-103a-3p | - | 0.9339 (0.8810-0.9868) | 88.0 | 95.1 | 44 | 97 | 5 | 6 |
|  |  |  |  |  |  |  |  |  |
| Men ≤ 60 years of age | | | | | | | | |
| Mesothelin [nmol/L] | 1.250 | 0.8994 (0.8244-0.9745) | 76.9 | 94.8 | 30 | 73 | 4 | 9 |
| Calretinin [ng/ml] | 0.414 | 0.9422 (0.8950-0.9894) | 84.6 | 94.8 | 33 | 73 | 4 | 6 |
| miR-103a-3p (normalized) | 1438.152 | 0.5361 (0.4261-0.6461) | 12.8 | 94.8 | 5 | 73 | 4 | 34 |
| Mesothelin, calretinin, miR-103a-3p | - | 0.9467 (0.8956-0.9978) | 87.2 | 94.8 | 34 | 73 | 4 | 5 |
| Mesothelin and calretinin | - | 0.9497 (0.9017-0.9977 | 84.6 | 94.8 | 33 | 73 | 4 | 6 |
| Mesothelin and miR-103a-3p | - | 0.9004 (0.8249-0.9759) | 76.9 | 94.8 | 30 | 73 | 4 | 9 |
| Calretinin and miR-103a-3p | - | 0.9417 (0.8936-0.9898) | 84.6 | 94.8 | 33 | 73 | 4 | 6 |
